# Proper Care of Poor Children’s Teeth

**Published:** 1886-10

**Authors:** J. Hooper

**Affiliations:** Louisville


### Proper Care of Poor Children's Teeth.

(Read before the Kentucky State Dental Society.)

J. HOOPER, LOUISVILLE.

The subject proposed for consideration, and one that should interest the entire civilized world, I purpose to discuss as follows:

First. The relation which the children of the poor sustain to the public at large. The parents and grandparents of the poorer classes of children, have the same affection for their offspring, as the wealthy. The poor widow, so frequently left with a large family of children, without means to procure the necessaries of life for their support, dependent upon public charity, feels the same for them as does her more fortunate sister blessed with all the luxuries of life. The orphan, devoid of the care of a father's love, and a mother's self-sacrificing affection, is a class which clamors for our earnest attention, and shall their appeals to us meet no response? We all have poor unfortunate friends who have children, and being taken away, have left them without means sufficient for their needs.

Gentlemen, see our own profession; should reverses of fortune, sickness, or death, visit us to-day, how soon might our widows and children be cast forth upon the charity of the public?

Shall we stand over patients from day to day, spend our leisure hours in study, attend associations, and exchange views for mutual improvement, that we may labor for the few who will care for their teeth, and neglect the large number unprovided for, by failing to arouse and interest the public in this great cause, by showing them the duty they owe this class of unfortunates?

We cannot be true to the science of dentistry, gentlemen, if we fail to make an effort in so noble a cause.

Second. The design of teeth.

An All-Wise Power bestowed upon our race twenty deciduous teeth, thirty-two permanent teeth for the purpose of masticating the food, essential for our development and sustenance. The physician will assert that without the proper mastication of food, the foundation for indigestion and many diseases is laid which prevents growth in childhood, retards education, and entails many ills which "flesh is heir to." A healthy state of the teeth often insures success to the man of business. The clerk, the professional man, and the soldier have all prospered by it. Hannibal, Napoleon, Wellington, and Lee attribute their greatest defeats to indigestion, which was produced by non-mastication of food. How many business men that have failed to prosper, may not have so done from the same cause? How many book-keepers and clerks have made their employers lose largely and frequently, and probably lost their positions from the same cause? The teacher who instructs the youth of our land, the physician who frequently holds life in his hands and should have a clear unclouded brain, the lawyer, the judge may all be sufferers from it. Think of it! Life, success, everything depends upon mastication and assimilation. How important then that a perfect foundation be laid in the saving and care of the little one's teeth! The teeth are more valuable than diamonds. The first teeth are of more value than is generally supposed from the fact that they lay the foundation for the permanent teeth which have such an influence on our after life. Many cases are reported where diseases of children's teeth have produced diseases of the eye and ear. Dr. Samuel Sexton, of New York, has proven by the examination of quite a number of children in the various institutions of that city, that the above assertion is true. Any scientific oculist will attest the same truth. See, then, what an influence the teeth have on man's system, and how important that they be well cared for. The strong brave man, that would face the cannon's mouth without flinching, is the veriest whining babe with the tooth-ache. I believe that many chronic cases of disease are occasioned by the neglect of children's first teeth. If a child's teeth hurt it, a failure to masticate its food will result therefrom. This may continue through months and years, and habits be formed which may follow the child throughout life. What a responsibility rests upon parents and dentists!

Third. Gentlemen, what are we as a profession, state, or nation, doing to advance the cause, the proper care of poor children's teeth?

The definition of the word "Dentistry" signifies treatment of diseases of the mouth and teeth, filling and saving of the teeth. Statistics testify to the truth that the enormous sum of 22,000,-000 teeth are annually extracted in America; that the "land of the free and the brave" should tolerate such slavery from the forceps appears incredible. How can we, belonging to a scientific profession, whose mission it is to save the teeth, allow the sacrifice of such valuable organs and enter no protest against it.

In reading a little article from the *Medical Review*, headed, "Choice of Teeth for Extraction for the purpose of Uniformity," I see where one Dr. P. has stated in order to throw some light on the subject; that of 7277 permanent teeth extracted, it was found that 2823 were first permanent molars, 737 were first bicuspids, and 944 were second bicuspids, and further states, as statistics show, that "more first molars are lost than bicuspids, we should, as a rule, extract the first molars." I venture to assert that man destroyed more teeth than he saved.

As dentistry means the salvation of the teeth, by an overruling majority he would be classed as an anti-dentist.

There is another class who make the same mistake, and should be deemed opposed to the science, although they bear the name of dentists. I refer to those who with flashy cards and signs will advertise: "Pure Gold Fillings For One Dollar, Full Set of Teeth so much, Teeth Filled Without Pain, Teeth Inserted Without Plates, Straightening Teeth a Specialty, Pure Gas for the Painless Extraction of Teeth," and other modes too numerous to mention. The specialty of destroying the teeth is more applicable in their cases

What would we say, gentlemen, of the physician who would thus advertise with his cards and signs, "Pure Chloroform, Ether, or Cocaine for the painless cutting off of limbs and removing eyes? Would not his professional brethren at once cry, "Charlatan! Quack! Imposter!" And the public say, "police arrest him, we will not allow our unfortunate citizens to be misled in such a manner."

The press, ever ready to espouse the cause of the poor and afflicted, would denounce such an one in the boldest terms. Yet, we in our profession sit idly down and permit the gross mistakes to which I have referred.

I have a few casts here to show what serious deformities occur by the extraction of teeth, especially among children. If we extract a tooth the cavity will in course of time become closed, or if we file teeth they will come together again unless a shoulder is left. When teeth are filed, as they usually are, with a flat surface, the form of the tooth filed in this manner is changed, hence unnatural.

Here is a cast of a child seven years of age who had several teeth extracted four years ago, when but three years of age, which should have remained until she was nine or ten, that the jaw might grow with the child. Observe the deformity, please, gentlemen; see the contraction, and how it must disfigure her. Could you observe the little shrunken face, the appearance now of an old toothless woman, you would pity her. When she cuts her permanent teeth she will be classed with the irregulars.

I have had a case of a lady who brought her little daughter to me from a distance. She was sent by her dentist who extracted both of her upper six year or first molars about ten months previous. In that short space the upper arch contracted so that the under teeth protruded nearly half an inch beyond the upper teeth when the mouth was closed.

Here is another cast where a young man had his cuspid or eye teeth extracted by a dentist who informed him they were tusks. He was quite small then, but has grown to manhood now—twenty-two years of age. A cast of my little daughter's teeth at three years of age is the same size. Here is another cast of a gentleman who had a bicuspid extracted, and that side of the mouth is the same size of the child's.

I could cite others, but this is sufficient to show the mistakes of dentists and the deformities of the patients, and they not of the poorer classes. I have quite a number of casts of children's mouths, who were disfigured by the extraction of teeth. The center of the mouth being drawn to one side. The arch of the mouth in such cases meets the fate of the arch where brick or stone are removed—there is no perfect arch! The articulation and mastication are both interfered with when the arch is made imperfect.

Gentlemen, there should be more discretion used in the extraction of teeth, and more teeth treated and saved. How careful the good surgeon, before he amputates a limb, or takes out an eye. It is really terrible to think of the good teeth sacrificed daily without an effort being made to save them.

Gentlemen, we should treat our patients as we would desire our own family treated. I have visited several charitable institutions and inquired of quite a number of others, and find that but little has been done for the teeth of these little ones, except extract them, and find in a few instances where some dentist has filled teeth for some of the larger children, but such a limited number, that, as a profession, we could claim no credit for our charity. The States have done nothing in this. See how other charities have been aided, such as institutions for the blind, insane, feeble-minded, and deaf and dumb. What are we doing as a profession to assist a noble charity in our line? Nothing! nothing!!

The West Point Military Academy requires good teeth of her students, and I have been informed that two disfigured teeth would reject a student. The importance is realized in military schools, why should it not be in all other schools and business? Both public and private schools should be posted on this subject. I think it important in business also, as I have shown how illy we are fitted to attend to it, unless our condition is such that digestion is perfect.

The question is, how are we to care for the poor children's teeth? We can lend our aid by educating the public to know the value of the teeth, the necessity of caring for them. The press will greatly assist, enlist it in the great cause, and the consequence will be that cities, states, and nations must at last yield to its demands.

The work could be accomplished through the country as well as the cities. I have had gentlemen of means in discussing this subject with me, say that if by no other method than by taxation, they would acquiesce for the sake of the cause. You see if the public be rightly informed, there will be no difficulty in procuring means for the accomplishment of the work.

There is not time to discuss ways and means now.

